# On the Improved Mode of Making Vegetable Extracts

**Published:** 1815-10

**Authors:** Richard Battley

**Affiliations:** of Fore-Street, London.


*For the London Medical and Physical Journal.*

### *On the improved Mode of making Vegetable Extracts;* by RICHARD BATTLEY, Esq. of Fore-street, London.

AFTER considerable attention given to the preparation of narcotic medicines, especially conium, digitalis, and belladonna, I submitted to the College of Physicians specimens of this class of vegetables, so preserved in leaf, powder, and extract, as to retain all the active virtues of the medicines.

The censors of the college, through their registrar, Dr. Cope, were pleased to favor me with a reply, stating that they much approved of the specimens, and had ordered them to be deposited in the collection of the materia medica of the college.

I am further induced by the handsome manner in which Dr. Powell, in the translation of the Pharmacopœia lately published, has mentioned my efforts to serve the profession with these medicines in their most efficient form, to communicate, through your Journal, the methods by which I have accomplished so desirable a purpose.

Plants, which from circumstances cannot be operated upon immediately after they are cut, must be revived by immersing their stalks in water for twelve or eighteen hours; such as are perfectly recovered, which will be known by the leaves becoming as fresh as when growing, are to be bruised and pressed, and the juice from them passed through a fine hair sieve, and immediately placed on the fire. Some time before the boiling temperature takes place, a quantity of green-coloured matter begins to float on the surface of the fluid; in some plants this matter is very considerable, which is to be carefully removed by means of a thin perforated tin-dish. By the time the liquor boils, or soon afterwards, this green matter ceases to appear. The boiling is to be continued until til somewhat better than half the fluid has been evaporated, when the decoction is to be put into a conical pan and suffered to stand in it until cold. A large precipitation of dark green-coloured matter will be found to have taken place, the fluid swimming over which is to be again exposed to evaporation until half of it is consumed, and then placed again in the situation of precipitation. The precipitated matter from the second coction is by no means so green as the first. The remaining fluid is suffered to boil till it acquires the consistency of syrup, when the matters which had been collected at its commencement, by filtration and precipitation, are to be mixed with it and placed in a metallic pan in a water-bath. From this moment the operator must give his constant attention to the process till it is finished. It is not necessary that the matter should be kept constantly stirring, but the operator ought to have this strongly in his mind, viz. never to suffer the matter operated on to stick or become hard to the sides of the pan; if allowed so to harden, the extract loses its green colour, and in proportion to such loss is the deterioration of the medicine.

Previous to the process of drying the leaves of plants, the same rules must be carefully observed in reviving them, that were recommended previous to their being pressed for extract. The leaves, being in a high state of preservation and entirely freed from the stalks, and as much as possible from external moisture, are laid in thin layers on willow baskets, stripped of their bark, in a drying-room, from which the light is quite excluded, to a temperature of not less than from 130 to 140 degrees of Fahrenheit's thermometer, for three or four hours, or until the leaves begin to shrivel; the leaves are then to be turned in the same temperatuie, and the heat kept up for six or eight hours longer, when the operation is generally finished, which is known by the leaves crumbling without much difficulty in the hand.

If the process has been in all its parts properly managed, the result will be a beautiful green preparation, possessing in a high degree the medical properties of the plant. To preserve them in this desirable state, oil-jars, made perfectly clean and dry, are found to answer best; the leaves are to be lightly placed in the jar, the mouth of which is to be closely cemented. The jars so filled ought to be kept in a dry and warm situation.*

*August*, 1815.

* When gentlemen send transcripts of their favours to contemporary Journals, we request the favour of them to apprize us of the same, as it is not our wish to seem to copy from other works. —Edit.

